# RE: Water pipe (shisha) smoking among male students of medical colleges in the eastern region of Saudi Arabia

**DOI:** 10.4103/0256-4947.65260

**Published:** 2010

**Authors:** Jayadevan Sreedharan

**Affiliations:** Assistant Director and Associate Professor of Biostatistics, Research Division, Gulf Medical University, Ajman, United Arab Emirates drjayadevans@gmail.com

**To the Editor:** This is to bring to your kind attention the data analysis in this article.^1^ In the results section the author mentioned the prevalence of shisha smoking as 12.6% (47 out of 371) whereas in the discussion the overall prevalence of shisha and cigarette (only 15 using both and 58 using shisha only/cigarette only/shisha and cigarette) smoking as 12.6%. It is contradictory.

In Table 3, with regard to mother’s education and shisha use, in calculating the association, nonrespondents are also included in the Chi-square test. No explanation given in the text. While calculating the prevalence of shisha use among mothers with education, instead of the column total, the row total should have been taken as the denominator.

The last paragraph of the results section says a significantly higher proportion of shisha smokers were students from the College of Medicine compared to the other two colleges. In calculating the prevalence it is better to use the total number of students who participated in the study from each college as the denominator instead of the total number of tobacco users. Actually in this study more than a quarter of dental students (15/51) are shisha users whereas among medical students only 8.4% (19/225) are shisha users (Table 3 gives the wrong impression). Therefore, the higher prevalence of shisha use is among dental students and the least among the medical students.

## References

[1] Taha AZ, Sabra AA, Al-Mustafa ZZ, Al-Awami HR, Al-Khalaf MA, Al-Momen AA (2010). Water pipe (shisha) smoking among male students of medical colleges in the eastern region of Saudi Arabia. Ann Saudi Med.

